# P-1740. Impact of a System Wide Antimicrobial Stewardship Intervention Bundle to Improve Guideline Directed Antibiotics for Urinary Tract Infections

**DOI:** 10.1093/ofid/ofae631.1903

**Published:** 2025-01-29

**Authors:** Erin Weslander, Jaime Borkowski, William Justin Moore, Christie M Bertram, Nathaniel J Rhodes, Stephanie Chang, Rishita Shah, Aaron Oliver, Michael Dickens, Kyle Johnicker, Radhika S Polisetty, Jong Park, Sarah Sutton, Michael Postelnick

**Affiliations:** Northwestern Memorial Hospital, Chicago, Illinois; NM Delnor Hospital, Geneva, Illinois; Northwestern Medicine, Chicago, Illinois; Northwestern Memorial Hospital/Rosalind Franklin University of Medicine and Science, Chicago, Illinois; Midwestern University, Downers Grove, IL; Northwestern Medicine - Huntley Hospital, Huntley, Illinois; Northwestern Lake Forest Hospital, Lake Forest, IL, Illinois; Northwestern Medicine, Chicago, Illinois; Northwestern Medicine, Chicago, Illinois; Northwestern Medicine Kishwaukee Hospital, DeKalb, Illinois; Midwestern University College of Pharmacy/ Northwestern Medicine Central DuPage Hospital, Winfield, Illinois; Northwestern Medicine, Chicago, Illinois; Northwestern University, Chicago, Illinois; Northwestern Medicine, Chicago, Illinois

## Abstract

**Background:**

Urinary tract infections (UTIs) are one of the common reasons for antibiotic use across healthcare settings. Across an eleven-hospital system, common urinary pathogens (*E. coli*, *Proteus* species, and *Klebsiella* species) had greater than 80% coverage with cefazolin, which was within 0-8% of ceftriaxone coverage based on the local antibiograms. With increased risk of *C. difficile* infection and resistance development seen with ceftriaxone, cefazolin was promoted as first line for empiric UTI coverage.

**Table 1:**
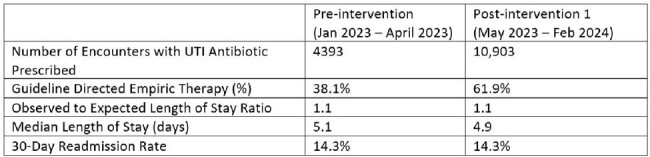
Outcomes

**Methods:**

An interrupted time series analysis was performed comparing a pre-intervention group to two phases of stewardship intervention (figure 1). The first intervention included an updated guideline, education, and pharmacist intervention upon order verification recommending guideline directed therapy. Guideline directed options and guideline non-compliant options are highlighted on green and red respectively, on figure 3. The second intervention was cascade reporting (figure 2) for urine culture Enterobacterales susceptibility, which was implemented across sites between 9/2023-1/2024. A generalized linear model with Poisson link was fit to the data. Compliant orders were regressed vs. the denominator of all UTI orders. Multiple models were considered, and the final model selection was based on likelihood ratio testing and the rule of parsimony.

**Figure 1:**
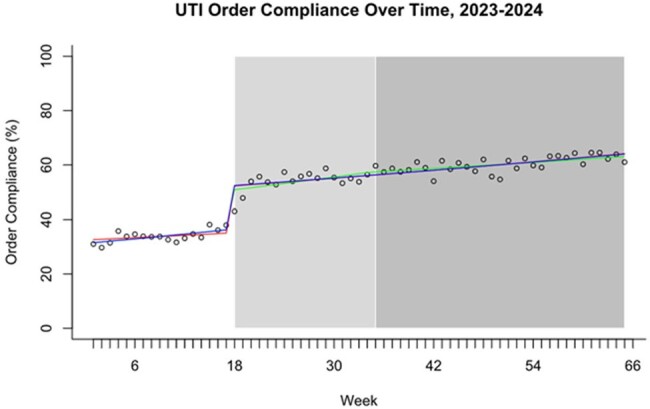
Interrupted Time-Series White = pre-intervention; light gray = intervention 1; dark gray = intervention 2

**Results:**

In the four pre-intervention months, guideline compliance across the system was 38.1% (1674/4393), compared to 61.9% (6749/10,903) after intervention 1 was implemented (table 1). The final model suggested that intervention 1 was associated with a nearly 1.5-fold increase in compliance which was sustained over time and suggested that the compliance rate over time increased modestly at around 5% per quarter (figure 1). There was no change in length of stay, risk adjusted length of stay, or 30-day all-cause readmission (table 1).

**Figure 2:**
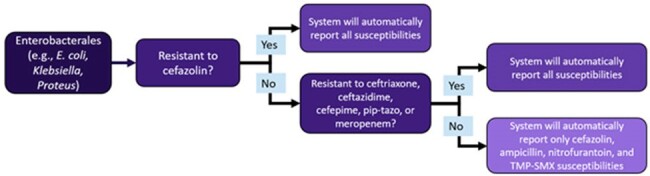
Microbiology Cascade Reporting Workflow

**Conclusion:**

Guideline update, education, and frontline pharmacist intervention were significantly impactful in improving guideline directed treatment for UTIs. Cascade reporting on urine cultures may help reinforce guideline directed therapy but did not result in substantially different compliance rates from education with frontline pharmacist intervention.

**Figure 3:**
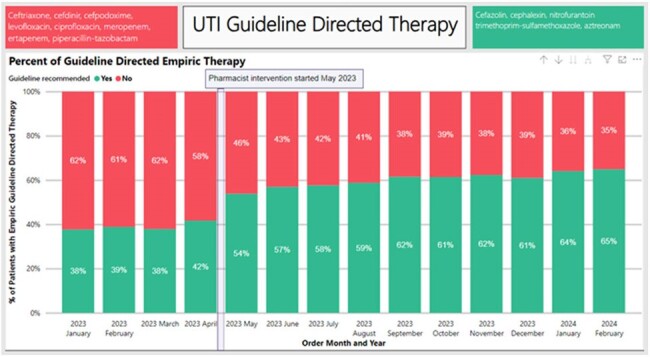
Percent of Patients with Empiric Guideline Directed Therapy Orders

**Disclosures:**

**Nathaniel J. Rhodes, PharmD MS**, Apothecademy, LLC: Advisor/Consultant

